# Multimodality Imaging in the Diagnosis of Prosthetic Valve Endocarditis: A Brief Review

**DOI:** 10.3389/fcvm.2021.750573

**Published:** 2021-12-20

**Authors:** Maxwell D. Eder, Krishna Upadhyaya, Jakob Park, Matthew Ringer, Maricar Malinis, Bryan D. Young, Lissa Sugeng, David J. Hur

**Affiliations:** ^1^Section of Cardiovascular Medicine, Yale University School of Medicine, New Haven, CT, United States; ^2^Ascension Medical Group, Section of Cardiovascular Medicine, Milwaukee, WI, United States; ^3^Department of Internal Medicine, Yale New Haven Hospital, New Haven, CT, United States; ^4^Section of Infectious Diseases, Yale University School of Medicine, New Haven, CT, United States

**Keywords:** prosthetic, valve, endocarditis (infectious), multimodality, imaging, endocarditis team

## Abstract

Infective endocarditis is a common and treatable condition that carries a high mortality rate. Currently the workup of infective endocarditis relies on the integration of clinical, microbiological and echocardiographic data through the use of the modified Duke criteria (MDC). However, in cases of prosthetic valve endocarditis (PVE) echocardiography can be normal or non-diagnostic in a high proportion of cases leading to decreased sensitivity for the MDC. Evolving multimodality imaging techniques including leukocyte scintigraphy (white blood cell imaging), ^18^F-fluorodeoxyglucose positron emission tomography (FDG-PET), multidetector computed tomographic angiography (MDCTA), and cardiac magnetic resonance imaging (CMRI) may each augment the standard workup of PVE and increase diagnostic accuracy. While further studies are necessary to clarify the ideal role for each of these imaging techniques, nevertheless, these modalities hold promise in determining the diagnosis, prognosis, and care of PVE. We start by presenting a clinical vignette, then evidence supporting various modality strategies, balanced by limitations, and review of formal guidelines, when available. The article ends with the authors' summary of future directions and case conclusion.

## Clinical Vignette

A 61-year-old man is brought to the emergency department with chief concerns of fever and confusion. Medical history is notable for bicuspid aortic stenosis status post bioprosthetic aortic valve replacement 3 months prior to presentation with his postoperative course complicated by sternal wound infection. In the emergency department he is noted to be febrile, tachycardic, and hypotensive. He is treated for septic shock and blood cultures subsequently grew methicillin-susceptible *Staphylococcus aureus* on day of presentation. Despite treatment with oxacillin, over the next 2 days he continues to be bacteremic with progressive PR prolongation to 240 ms noted on serial electrocardiograms. Given the high clinical concern for prosthetic aortic valve endocarditis with root abscess the patient undergoes transesophageal echocardiogram 2 days after initial positive blood cultures but does not reveal any abscess or vegetation (Supplementary Table 1 and Supplementary Figure 1A). How should this patient be further managed?

## Introduction

Infective endocarditis (IE) is an increasingly common infectious disease with incidence rates in the United States rising from 11 per 100,000 to 15 per 100,000 between 2000 and 2011 (1, 2). The disease also carries a high mortality rate with 5-year mortality of ~40% and in-hospital mortality ranging from 15 to 22% (3–5). Patients with IE require early diagnosis given both the treatable nature of this condition and potential complications of delayed antibiotics and surgery.

In 1994, the original Duke criteria were published by Durack et al. to facilitate the diagnosis of IE (6). However, the extrapolation of these criteria to real-world clinical patients remained challenging. In 2000, citing the need to redefine “possible IE,” and improve the ODC's sensitivity in the detection of Q fever, Li et al. established the modified Duke criteria (MDC; Supplementary Table 2) by proposing several changes to the existing major and minor criteria including further strengthening the role of echocardiography and narrowing the definition of “possible IE” (7). Subsequently, the MDC through the combined use of clinical, echocardiographic, pathologic, and microbiological data have since become one of the most widely used clinical tools for the detection of IE.

However, in cases of prosthetic valve endocarditis (PVE), echocardiography can be normal or non-diagnostic in around 30% of cases, leading to reduced diagnostic accuracy for the MDC (8, 9). This can be a particularly vexing issue, given the increasing use of intracardiac prosthetic materials, and the relatively high proportion of prosthetic material associated with endocarditis (10–13). Fortunately, newer and novel approaches to cardiac imaging including leukocyte scintigraphy (white blood cell imaging), ^18^F-fluorodeoxyglucose positron emission tomography (FDG-PET), multidetector computed tomographic angiography (MDCTA), and cardiac magnetic resonance imaging (CMRI) can be considered as potential adjunct tools in the evaluation of suspected PVE (14). The various modalities' advantages, limitations, characteristics, and other considerations are highlighted in Table 1. The various modalities' recommended applications as they pertain to recent clinical guidelines (13, 15) are outlined in Table 2.

**Table 1:** Overview of imaging modalities in the detection of prosthetic valve endocarditis.

**Imaging Modality**	**Advantages**	**Limitations**	**Test characteristics**	**Other considerations**
TTE	• Non-invasive • Fast and cost effective • Provides both functional and anatomic data • Accessible technology/can be performed at bedside • Does not use radiation	• Limited sensitivity in PVE • Limited sensitivity in detecting abscesses and paravalvular involvement • Unable to assess for extracardiac manifestations	• Sensitivity for NVE 50–90% • Specificity for NVE >90% • Sensitivity for PVE 36–69%	• Useful and cost effective first line test for suspected IE
TEE	• Improved sensitivity over TTE for NVE and PVE • Provides both functional and anatomic data • Can be performed at bedside • Does not use radiation	• Semi-invasive • Procedural risks including sedation-related, aspiration, aerosolization, oropharyngeal-esophageal injury • Reduced sensitivity in PVE compared to NVE • Limited sensitivity in detecting abscesses and paravalvular involvement, possibly earlier on in disease course • Unable to assess for extracardiac manifestations	• Sensitivity for NVE of 90–100% • Sensitivity for PVE 82–96% • Specificity for IE 92–95%	• Appropriate second test if TTE is negative or inconclusive and clinical suspicion remains high
Leukocyte Scintigraphy	• High specificity for infection • Ability to assess paravalvular complications • Ability to assess extracardiac manifestations • Relatively wide availability (compared to PET/CT) and low cost	• Decreased sensitivity for detection of vegetations • Labor intensive, requires multiple sessions • Radiation exposure	• Sensitivity for IE 64–90% Specificity for IE 100% • Sensitivity for Abscess 83–100% • Specificity for Abscess 78–87%	• Useful test when high specificity is desired or for examining extracardiac manifestations of IE
FDG-PET	• High sensitivity in PVE • Enhanced anatomic resolution relative to leukocyte scintigraphy • Ability to assess paravalvular complications • Ability to assess for extracardiac manifestations	• Lower specificity—non-infectious inflammation can lead to false positives • Limited sensitivity in NVE • Radiation exposure • Dietary restrictions necessary for preparation	• Sensitivity for IE 73–100% • Specificity for IE 71–100%	• Useful test to follow a non-diagnostic TEE when clinical suspicion for PVE remains high
MDCTA	• Provides detailed anatomic data on coronary vasculature and valvular anatomy which can aid in perioperative planning • High sensitivity for paravalvular complications	• Limited ability to detect valve perforations and dehiscence • Limited ability to detect small vegetations • Risk of contrast induced nephropathy • Radiation exposure	• Sensitivity for IE 93–100% • Specificity for IE 83–97%	• May be ideal when both diagnostic and perioperative anatomic data are needed • Performance may be optimal when paired with tests with functional information such as echocardiography or FDG-PET
CMRI	• Provides highly detailed anatomic and functional data • May offer sensitivity to detect even small vegetations • Does not use radiation	• Not well-studied for detection of IE and limited data on ideal application • Artifacts or incompatibility from mechanical/ferromagnetic implants	• Limited data	• Further data is needed to clarify the role of this rapidly evolving modality

*CMRI, cardiac magnetic resonance imaging; FDG-PET, ^18^F-fluorodeoxyglucose positron emission tomography; IE, infective endocarditis; MDCTA, multidetector computed tomographic angiography; NVE, native valve endocarditis; PVE, prosthetic valve endocarditis; TEE, transesophageal echocardiogram; TTE, transthoracic echocardiogram*.

**Table 2:** Imaging modalities and infective endocarditis guidelines.

**Imaging Modality**	**2015 European Society of Cardiology Guidelines for the Management of Infective Endocarditis**	**2020 American College of Cardiology/American Heart Association Guidelines for the Management of Patients with Valvular Heart Disease**
TTE	• TTE is recommended as the first line imaging modality in suspected IE (class I, level of evidence B) • Repeat TTE and/or TEE within 5–7 days is recommended in case of initially negative examination when clinical suspicion of IE remains high (class I, level of evidence B)	• In patients with suspected IE, TTE is recommended to identify vegetations, characterize the hemodynamic severity of valvular lesions, assess ventricular function and pulmonary pressures, and detect complications (class I, level of evidence B-NR)
TEE	• TEE is recommended in all patients with a clinical suspicion of IE and a negative or non-diagnostic TTE (class I, level of evidence B) • TEE should be considered in patients with suspected IE, even in cases with positive TTE, except in isolated right-sided NVE with good quality TTE examination and unequivocal echocardiographic findings (class IIa, level of evidence C)	• In all patients with known or suspected IE and non-diagnostic TTE results, when complications have developed or are clinically suspected or when intracardiac device leads are present, TEE is recommended (class I, level of evidence B-NR) • In patients with a prosthetic valve in the presence of persistent fever without bacteremia or a new murmur, a TEE is reasonable to aid in the diagnosis of IE (class IIa, level of evidence B-NR)
Leukocyte Scintigraphy	• Leukocyte scintigraphy should be preferred in situations that require increased specificity given the modality is more specific for the detection of IE and infectious foci than FDG-PET	• No specific recommendation
FDG-PET	• Advantages of FDG-PET include reducing the rate of misdiagnosed IE by reducing those classified as possible IE via the Duke criteria and detection of metastatic and peripheral infections or embolic events • Limitations to use include localization of cerebral septic emboli due to high physiologic uptake in the brain, and low spatial resolution of current PET/CT scanners • Caution should be used when interpreting patients who have undergone recent CT surgery	• In patients classified by Modified Duke Criteria as having “possible IE,” FDG-PET/CT is reasonable as adjunct diagnostic imaging (class IIa, level of evidence B-NR)
MDCTA	• For the evaluation of PVE MDCTA may perform similarly or even superiorly to echocardiography when it comes to the detection of prosthesis associated dehiscence, vegetations, abscesses, and pseudoaneurysms. However, due to a lack of large comparative studies between the two echocardiography should always be performed first	• In patients in whom the anatomy cannot be clearly delineated by echocardiography in the setting of suspected paravalvular infections, CT imaging is reasonable (class IIa, level of evidence B-NR)
CMRI	• Myocarditis and myocardial involvement may be best assessed using CMRI and TTE	• No specific recommendation

*Recommendations are quoted from the respective guidelines or summarized as appropriate. CMRI, cardiac magnetic resonance imaging; FDG-PET, ^18^F-fluorodeoxyglucose positron emission tomography; IE, infective endocarditis; MDCTA, multidetector computed tomographic angiography; NVE, native valve endocarditis; PVE, prosthetic valve endocarditis; TEE, transesophageal echocardiogram; TTE, transthoracic echocardiogram*.

## Leukocyte Scintigraphy (White Blood Cell Imaging)

Leukocyte scintigraphy can be highly specific for infection as it allows examination for the pathologic accumulation of radiolabeled granulocytes at involved sites through the use of single photon emission computed tomography (SPECT)/CT. Thus, leukocyte scintigraphy may offer high specificity (100%) for paravalvular infection and abscess detection in patients with suspected PVE (13, 14, 16). Furthermore, this imaging technique allows for evaluation of extracardiac manifestations of PVE, including endovascular infections, ophthalmitis, or intracranial infections, providing a broader picture of both cardiac and non-cardiac sources of infection (17, 18). The modality may yield not as much non-specific radiotracer uptake in the sternum in those who have recently (past 1 month) undergone cardiothoracic surgery, and may avoid confounding inflammation from certain other non-infectious pathologies (e.g., non-calcified atherosclerotic plaque, vasculitis, active thrombus, primary cardiac tumor or non-cardiac tumor that has metastasized to the heart, post-op inflammation, or foreign body reaction) that can mimic the pattern of FDG uptake seen with IE (19). Also, from an availability standpoint there may remain many centers that have SPECT/CT but not yet PET/CT available.

However, leukocyte scintigraphy also has potential limitations. For one, vegetations contain relatively few granulocytes, and therefore leukocyte scintigraphy may offer decreased sensitivity (64%) for the detection of PVE (14, 16). Furthermore, leukocyte scintigraphy offers decreased spatial resolution compared to other imaging modalities and is labor intensive requiring the drawing, preparation, and reinjection of granulocytes over multiple sessions. Currently, there is discordance between the European (13) and American (15) guidelines; white cell scans are recognized in the 2015 European Society of Cardiology (ESC) guideline for the management of IE as being more specific than FDG-PET, and ought to be preferred in clinical situations that would benefit from increased specificity, whereas the 2020 American College Cardiology (ACC)/American Heart Association (AHA) clinical practice valvular heart disease (VHD) guideline does not make a specific recommendation. While the ideal application of leukocyte scintigraphy in the detection of PVE may be currently unknown or debatable, this test may be best implemented when other modalities are inconclusive or when enhanced specificity is needed (13, 14, 16).

## ^18^F-fluorodeoxyglucose Positron Emission Tomography

Relative to leukocyte scintigraphy, FDG-PET offers several advantages: it is less labor intensive, has enhanced anatomical resolution, and offers increased sensitivity for the detection of PVE (14, 16, 20). For these reasons FDG-PET is increasingly used in difficult-to-diagnose cases of PVE where initial echocardiography is non-diagnostic. The presence of an abnormal signal in the region of interest in the valve's vicinity by FDG-PET was included as a major criterion for the diagnosis of PVE in the 2015 update (from 2009) ESC IE guideline (13). Adding FDG-PET as a major criterion to the MDC increases the sensitivity of the MDC from 52 to 70% up to 97% without sacrificing specificity, and aids in the early diagnosis of PVE, particularly when echocardiography is equivocal or normal (21, 22). A recent study by Primus et al. showed that FDG-PET improves diagnostic certainty when combined with MDC in both native and PVE (23). A contemporary meta-analysis of PVE or cardiac implantable electronic device (at least 3 months post-placement) endocarditis had a pooled sensitivity of 72–86% and specificity of 83–84% with use of FDG-PET (24). While no FDG uptake excludes the presence of PVE, an elevated ratio of FDG uptake at and around the prosthetic valve relative to background standardized uptake value (SUV) of >4.4 highly suggests PVE (16). Similar to leukocyte scintigraphy, FDG-PET offers the ability to detect distant emboli and foci of infection allowing for the characterization of extracardiac involvement, with the caveat that, due to high physiologic levels of FDG uptake in the brain, it may be limited in the detection of intracerebral infections (13, 20, 21, 25, 26). A related caveat is that cardiac physiologic uptake of glucose must be adequately suppressed with a high-fat, low-carbohydrate diet and prolonged fast prior to FDG-PET to reliably identify pathological uptake in structures adjacent to myocardium (27).

While FDG-PET is highly sensitive, it is less specific than leukocyte scintigraphy because FDG-PET uptake can be increased also in non-infectious sources of inflammation (14, 16). In particular, inflammation from recent cardiac surgery (within a month) has the potential to lead to false positive findings on FDG-PET, and therefore leukocyte scintigraphy with SPECT/CT or other imaging modalities may be preferred in these cases (13, 14, 17). The 2020 ACC/AHA VHD guideline gives FDG-PET a moderate strength (class 2a) recommendation when the MDC is possible IE (15). Due to the several advantages of this modality, including high sensitivity, feasibility, and ability to detect extracardiac involvement, it may be a logical choice to follow a negative or non-diagnostic transesophageal echocardiogram (TEE) when clinical suspicion for PVE remains high (13, 14, 21).

## Multidetector Computed Tomographic Angiography

While TEE, SPECT/CT, and FDG-PET provide functional data, MDCTA has the advantage of providing detailed anatomic images due to its high spatial resolution, with current scanners having the ability to have sub-millimeter (on the order of 0.5-mm) “isotropic” resolution in all three dimensions (28). With a full 3D cardiac dataset scan, post-processing in multiplanar reconstruction allows the valvular and prosthetic structures to be evaluated from any angle to fully interrogate valve and surrounding structures for precise anatomic assessment (29). In particular, MDCTA is adept in the detection of paravalvular abscesses, prosthetic dehiscence, and pseudoaneurysms, and may be better able to distinguish myocardial, pericardial, and coronary sinus involvement relative to TEE, while offering similar ability to detect non-highly mobile and larger vegetations (29–32). Additionally, due to the detailed anatomic information offered, as well as the ability to assess the status of the coronary arteries, MDCTA is particularly well-suited for perioperative evaluation and planning (13, 33). However, when compared with TEE, MDCTA may miss very small valve leaflet perforations and highly mobile vegetations due to the lower temporal resolution compared to echocardiography (30, 31). Therefore, as of the latest guidelines MDCTA may be best utilized as a complementary study to echocardiography, with current ESC guidelines for the management of IE suggesting that echocardiography should typically be performed first (13, 32, 34, 35). Additionally, the 2020 ACC/AHA clinical practice VHD guideline gives MDCTA a class 2a recommendation for suspected paravalvular abscess when echo images are inadequate (15). When added to the standard diagnostic workup including echocardiography, the addition of MDCTA improves sensitivity up to 100% and specificity of 83% (14). However, during the recent COVID-19 pandemic, in cases with risks of aerosolization with TEE, MDCTA has been seen as a reasonable alternative (29). Moreover, another avenue is to complement FDG-PET's functional information with MDCTA's anatomic data in order to maximize sensitivity, specificity, and diagnostic accuracy (22). As a result, while the use of MDCTA is not yet universal as an frontline test in PVE, there is growing data to support its earlier use in the diagnostic assessment in this patient group; a recent meta-analysis published after the 2020 ACC/AHA guideline showed that MDCTA performs better in identifying prosthetic valve infection and showed a trend of improved detection of para-annular complications of abscess and pseudoaneurysm formation compared to TEE (36). Thus, this modality offers promise in scenarios where detailed anatomic or perioperative data is needed to supplement the workup of PVE or as a complementary study to TEE or FDG-PET or even possibly as a frontline study.

## Cardiac Magnetic Resonance Imaging

Cardiac MRI is a rapidly evolving imaging modality that can provide both detailed anatomic as well as functional data on valvular regurgitation and the presence of myocardial edema and inflammation (14, 37, 38). When applied to patients with possible infective endocarditis, CMRI can identify valvular vegetations and paravalvular pseudoaneurysms, and detect the paravalvular extension of infection through the presence of delayed contrast enhancement (38–40). This led to the inclusion of CMRI as a new indication along with echocardiography to assess myocardial involvement during infective endocarditis within the subsection regarding complications of infective endocarditis relating to myopericarditis in the 2015 update of ESC guidelines from 2009 (41). Additional strides are being made recently in multimodality comparison between TEE and CMRI for the quantification of paravalvular regurgitation in left-sided (aortic or mitral) prosthetic valves (42). However, to date there are relatively few studies on the use of CMRI in the detection of PVE, and most of the existing data comes from small series or case reports (43, 44). Furthermore, CMRI carries potential limitations including valve-induced susceptibility artifacts with mechanical prosthetic valves, contraindications for patients with certain pacemakers and medical implants, and longer acquisition times (37). As a result, the ideal diagnostic role for CMRI in the workup of IE is still to be determined, and further studies are needed (14).

## Discussion

**Figure 1:**
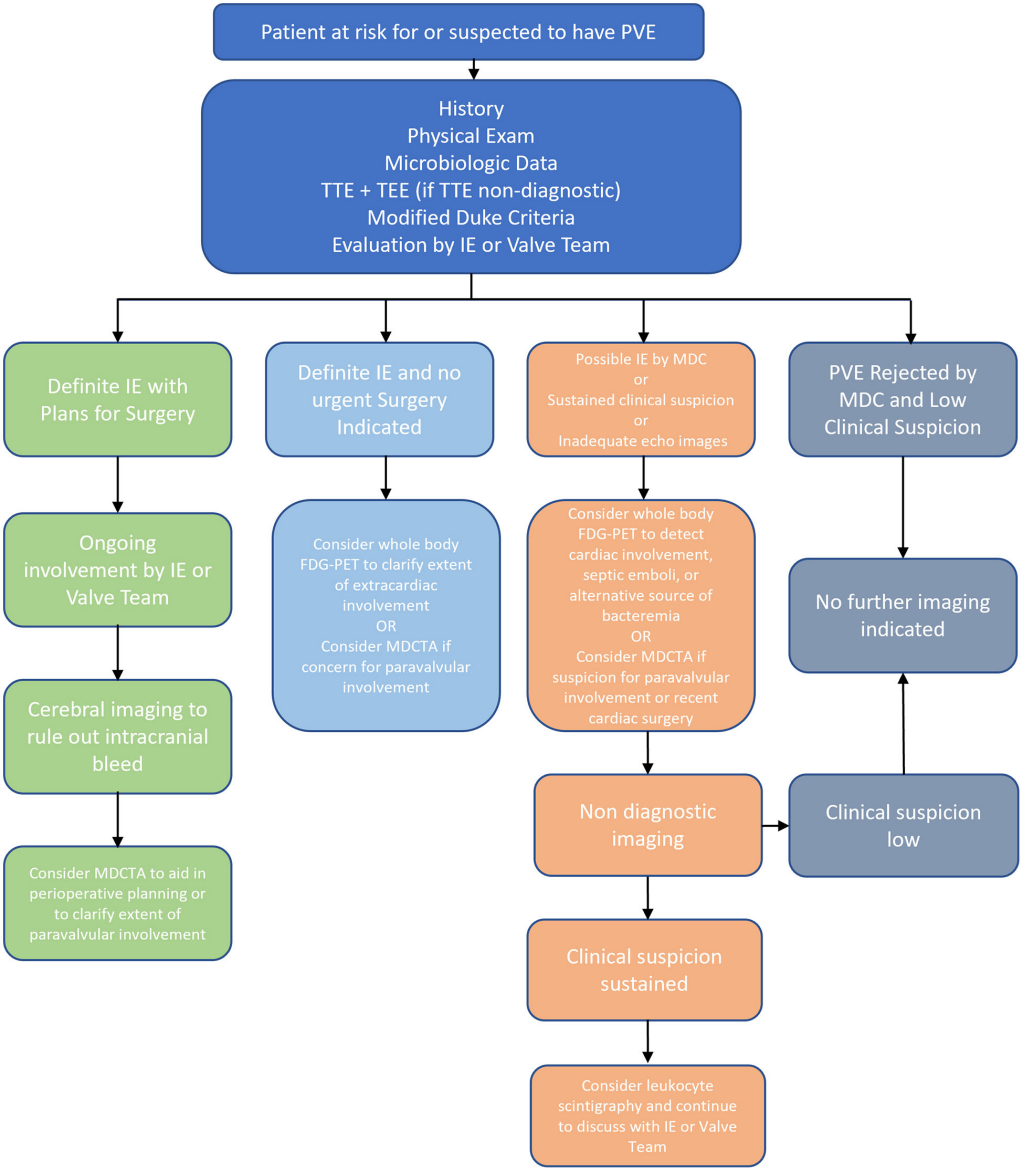
Overview of Diagnosis of PVE. FDG-PET, ^18^F-fluorodeoxyglucose positron emission tomography; IE, infective endocarditis; MDC, modified duke criteria; MDCTA, multidetector computed tomographic angiography; NVE, native valve endocarditis; PVE, prosthetic valve endocarditis; TEE, transesophageal echocardiogram; TTE, transthoracic echocardiogram.

PVE diagnosis remains a challenging and clinically important issue that is expected to be increasingly encountered given the rising use of intracardiac prosthetic materials. In cases of difficult-to-diagnose PVE, multimodality imaging techniques can provide utility beyond the standard diagnostic workup and serve to augment the MDC and echocardiography. Both leukocyte scintigraphy and FDG-PET offer the ability to evaluate for extracardiac involvement while carrying high specificity and sensitivity, respectively. Furthermore, MDCTA can provide key anatomic data, including paravalvular information, and is particularly useful for perioperative planning. While CMRI offers the possibility of detailed anatomic and functional data, currently there is insufficient evidence for the role of this modality in the routine workup of PVE and further studies are needed. The diagnostic approach to PVE is summarized in Figure 1.

While an important consideration is the cost effectiveness of each modality, unfortunately contemporary high-quality data in this area is currently lacking. Echocardiography is affordable and widely available although early studies of cost effectiveness in IE found it may be more cost effective to proceed with TEE as an initial diagnostic test in intermediate risk patients than to pursue sequential testing with transthoracic echocardiogram prior to TEE (45). Interestingly, in patients who already have a high pretest probability of IE, the most cost-effective strategy may be to simply pursue empiric treatment without additional echocardiographic imaging (45). Though more limited in availability, the addition of FDG-PET to detect metastatic infections in high-risk patients with Gram-positive bacteremia may be cost effective (46), however it is unclear if this would be true for the evaluation of endocarditis.

Costs and time to diagnosis increase with each sequential diagnostic test, and therefore strategies which reduce test stacking can improve both the cost effectiveness and time effectiveness of diagnosing PVE. Two strategies that are gaining attention are the use of diagnostic flowcharts to streamline the decision-making process, and the use of multimodality endocarditis teams to tailor the diagnostic workup. Contemporary evidence would suggest that diagnostic flow charts may be applicable to real-world clinical practice and may help increase diagnostic yield in PVE (47). However, the full use of complex diagnostic algorithms would require both the necessary imaging technology as well the appropriately specialized team to interpret the data and thus may be limited to certain hospital systems (48). A more individualized approach is the use of highly specialized and multidisciplinary endocarditis teams to guide diagnosis and treatment which may lead to earlier diagnosis and improved outcomes (49, 50). Through the use of these strategies, it may be possible to enhance diagnosis, prognosis, and patient care in PVE.

## Future Directions

Despite the utility of multimodality imaging tests, further larger prospective studies are needed to clarify the optimal role and refine the imaging protocol and interpretation of each of these tests in PVE diagnosis, as well as clarify the prognostic value of these tests.

More recently, within radionuclide imaging for PVE, studies have looked at better refining image acquisition time of FDG-PET (~60 min post intravenous injection as opposed to ~150 min) (51) to reduce late false-positive FDG uptake and utilizing SUVs (52). Uniform protocols and standardized metrics would facilitate the comparison of studies from various centers and support the creation of multicenter registries in this area of PVE imaging. The evolution of grading FDG uptake from just positive or negative to more qualitative (absent, mild, moderate, intense) grading, to eventual quantitation with the goal of developing SUV thresholds corresponding to each of the above qualitative levels of FDG uptake would aid to increase diagnostic precision and accuracy and relate these various levels to the probability of PVE in patients.

Currently, the diagnosis of PVE should continue to rely on clinical judgment, microbiological data, and echocardiographic studies with multimodality imaging augmenting this workup when additional sensitivity, specificity, functional data, or anatomic clarification is necessary. Analogous to how echocardiography is used in the modified Duke paradigm, the precise quantification of valvular FDG uptake SUVs could be how more major and minor criteria of PVE using nuclear imaging become delineated, perhaps in combination with the analysis of the pattern and location of FDG uptake in the valve and paravalvular regions of interest.

In the area of multidetector cross-sectional imaging with computed tomography, dual-energy scans, which can reduce beam hardening and partial volume averaging artifacts to improve tissue characterization quality via distinguishing between high- and low-photon energies, have shown some early promise, but thus far have been limited in clinical use (29). Spectral computed tomography may further delineate different tissue densities by utilizing multiple energy levels, and these modalities may aid in earlier vegetation and abscess identification while continued improvements in stent resolution and minimizations in artifacts with ultra-high-resolution scans (detector rows as low as 0.25 mm in width) may improve evaluation of the prostheses themselves.

## Conclusion

Following a non-diagnostic TEE, the patient in the clinical vignette underwent FDG-PET 4 days later, which revealed increased FDG uptake at the aortic valve prosthesis. Subsequent repeat TEE 3 days later and 1 week since original TEE revealed vegetations and an aortic root abscess (Supplementary Figures 1B-E and Supplementary Table 3). The patient was taken for redo aortic valve replacement with ascending aortic graft and root replacement. In summary, while PVE may provide a diagnostic challenge to the conventional workup of IE, multimodality imaging techniques such as leukocyte scintigraphy, FDG-PET, MDCTA, and CMRI can provide additional diagnostic value and aid in the correct diagnosis of PVE.

## Author Contributions

ME, LS, and DH devised the manuscript concept. ME collected articles and wrote the manuscript in collaboration with KU, JP, MR, and DH. JP, MR, MM, BY, LS, and DH critically reviewed and revised the manuscript. All authors approved the final manuscript.

## Conflict of Interest

The authors declare that the research was conducted in the absence of any commercial or financial relationships that could be construed as a potential conflict of interest.

## Publisher's Note

All claims expressed in this article are solely those of the authors and do not necessarily represent those of their affiliated organizations, or those of the publisher, the editors and the reviewers. Any product that may be evaluated in this article, or claim that may be made by its manufacturer, is not guaranteed or endorsed by the publisher.

## Abbreviations

ACC: American College of Cardiology
AHA: American Heart Association
CMRI: cardiac magnetic resonance imaging
ESC: European Society of Cardiology
FDG-PET: ^18^F-fluorodeoxyglucose positron emission tomography
IE: infective endocarditis
MDC: modified Duke criteria
MDCTA: multidetector computed tomographic angiography
PVE: prosthetic valve endocarditis
SPECT/CT: single photon emission computed tomography
SUV: standardized uptake value
TEE: transesophageal echocardiogram
VHD: valvular heart disease.
