# Supplementary material for: Multimodality Imaging in the Diagnosis of Prosthetic Valve Endocarditis: A Brief Review
Source: Front Cardiovasc Med. 2021 Dec 20;8:750573. doi: 10.3389/fcvm.2021.750573 (PMC8720921; doi:10.3389/fcvm.2021.750573)
Supplement: Supplementary file 1 [file Table_1.pdf]

Patient Characteristics and Initial Diagnostic Workup	Value (Reference Range) or Interpretation
Age (years)	61
Sex	Male
Heart rate (beats per minute)	109 (60 – 100)
Temperature (degrees Fahrenheit)	102.2 (97.0 – 99.0)
Blood Pressure (mmHg)	79/51 (90 – 120/<80)
Oxygen Saturation by pulse oximeter (%)	98% on 3 liters oxygen by nasal cannula
Sodium (mmol/L)	127 (136 – 144)
Potassium (mmol/L)	4.2 (3.3 – 5.1)
Chloride (mmol/L)	92 (98 – 107)
Carbon Dioxide (mmol/L)	15 (20 – 30)
Urea nitrogen (mg/dL)	42 (8 – 23)
Creatinine (mg/dL)	2.7 (0.40 – 1.30)
Glucose (mg/dL)	145 (70 – 100)
Calcium (mg/dL)	8.3 (8.8 – 10.2)
Total Bilirubin (mg/dL)	0.6 (<1.2)
Alkaline Phosphatase (U/L)	104 (9 – 122)
Alanine Aminotransferase (U/L)	35 (6 – 34)
Aspartate Aminotransferase (U/L)	47 (11 – 33)
Albumin (g/dL)	3.3 (3.6 – 4.9)
White Blood Cell Count (per µl)	10,300 (4,000 – 10,000)
Hemoglobin (g/dL)	10.7 (12.0 – 18.0)
Hematocrit (%)	32.4 (37 – 52)
Platelets (per µl)	141,000 (140,000 – 440,000)
Troponin I (ng/mL)	0.57 (0.00 – 0.08)
Troponin T (ng/mL)	0.05 (<0.01)
Blood cultures on admissions (2 sets)	Aerobic and Anaerobic bottles growing methicillin susceptible *Staphylococcus aureus* (MSSA)
Electrocardiogram:	Sinus tachycardia with a rate of 107, left axis deviation, normal PR interval, interventricular conduction delay with right bundle branch block morphology, normal QTc interval with T wave inversions in V5 and V6 . Q waves are present in III and aVF. Overall similar in appearance to electrocardiogram prior to admission. No clear ischemic ST segment changes.
CT Chest without Intravenous Contrast:	Bilateral, right greater than left ground glass opacities that may represent infectious/inflammatory etiology. No focal consolidation is identified. Post-sternotomy changes as before with stable appearance of previously seen retrosternal fluid and fat stranding in the superior mediastinum.
Transthoracic Echocardiogram:	Mild left ventricular hypertrophy with severe global hypokinesis and LVEF of 27%. Dilated right ventricle with moderately decreased right ventricular systolic function. Prosthetic aortic valve appears to be functioning normally. Mild-moderate mitral regurgitation, mild-severe tricuspid regurgitation. Overall similar to echocardiogram 3 months prior to admission.
Transesophageal Echocardiogram #1:	Severely global hypokinesis and LVEF of 15-20%. Moderately decreased right ventricular systolic function. No evidence of thrombus in left atrial appendage, left atrium, right atrium or right atrial appendage. A 27mm bioprosthetic aortic valve is present and appears to be functioning normally without intravalvular or paravalvular regurgitation. Mild mitral regurgitation. Moderate tricuspid regurgitation.

Supplemental Table 1: Overview of clinical vignette patient's characteristics and initial diagnostic workup
