# Supplementary material for: Multimodality Imaging in the Diagnosis of Prosthetic Valve Endocarditis: A Brief Review
Source: Front Cardiovasc Med. 2021 Dec 20;8:750573. doi: 10.3389/fcvm.2021.750573 (PMC8720921; doi:10.3389/fcvm.2021.750573)
Supplement: Supplementary file 2 [file Table_2.pdf]

<table>
<tr><th>Pathological Criteria</th><th>Major Clinical Criteria</th><th>Minor Criteria</th></tr>
<tr><td rowspan="3">Microorganisms in a vegetation: Demonstrated by culture or histologic examination of a vegetation, a vegetation that has embolized, or an intracardiac abscess specimen.</td><td rowspan="3">Blood cultures positive for infective endocarditis: Typical microorganisms consistent with IE from 2 separate blood cultures, microorganisms consistent with IE from persistently positive blood cultures, single positive blood culture for Coxiella burnetii or antiphase I IgG antibody titer >1:800.</td><td>Predisposing heart condition or injection drug use</td></tr>
<tr><td>Fever: Defined as a temperature >38 degrees Celsius</td></tr>
<tr><td rowspan="2">Vascular phenomena: Major arterial emboli, septic pulmonary infarcts, mycotic aneurysm, intracranial hemorrhage, conjunctival hemorrhages, and Janeway’s lesions</td></tr>
<tr><td rowspan="3">Pathologic lesions: Vegetation or intracardiac abscess confirmed by histologic examination showing active endocarditis.</td><td rowspan="3">Evidence of endocardial involvement: Echocardiogram positive for IE: Oscillating intracardiac mass on valve or supporting structures, abscess, new partial dehiscence of prosthetic valve, or new valvular regurgitation.</td></tr>
<tr><td>Immunologic phenomena: Glomerulonephritis, Osler’s nodes, Roth’s spots, and rheumatoid factor</td></tr>
<tr><td>Microbiological evidence: Positive blood culture not meeting a major criterion, or serologic evidence of active infection with an organism consistent with IE</td></tr>
</table>

Supplemental Table 2: Modified Duke criteria

Adapted from Li et al, 2000. Abbreviation: IE = infective endocarditis. Definite IE: when either pathologic criterion is met; 2 major criteria are met; or 1 major criterion and 3 minor criteria are met; or when 5 minor criteria are met. Possible IE: 1 major and 1 minor criterion are met; or 3 minor criterion are met. Rejected: Firm alternate diagnosis; or resolution of IE syndrome with antibiotic therapy less than or equal to four days; or no pathologic evidence of infective endocarditis at surgery or autopsy with antibiotic therapy less than or equal to four days; or not meeting criteria for possible IE as noted above.
