# Supplementary material for: Multimodality Imaging in the Diagnosis of Prosthetic Valve Endocarditis: A Brief Review
Source: Front Cardiovasc Med. 2021 Dec 20;8:750573. doi: 10.3389/fcvm.2021.750573 (PMC8720921; doi:10.3389/fcvm.2021.750573)
Supplement: Supplementary file 3 [file Table_3.pdf]

Patient Subsequent Diagnostic Workup	Interpretation
Whole Body ^{18}F-fluorodeoxyglucose positron emission tomography:	Intense uptake at the aortic valve prosthesis concerning for infection. Nonspecific nodal, splenic, and bone marrow activity probably reactive. Lung changes probably non-infectious although left lower lobe infection cannot be excluded.
Transesophageal Echocardiogram #2:	A 27 mm Carpentier Edwards bioprosthetic aortic valve appears well-seated; however, there is evidence of vegetations and aortic root abscess. Multiple echodensities (up to 1.2 cm) consistent with vegetations seen on two of the aortic leaflets. Peak aortic velocity is 2.1 m/s. Mean gradient is 11 mmHg. Dimensionless index is 0.28. Trace aortic regurgitation. Hypoechoic thickening seen at 45 degrees posterior to the aortic valve in short axis view measuring 2.4 cm x 1.5 cm and at 135 degrees in the long axis view of the aortic valve in continuity with the anterior mitral valve leaflet measuring 1.3 cm x 2.4 cm that likely represents an aortic root abscess. Compared with earlier TEE study 7 days prior, there are significant changes. There is now a prosthetic valve abscess and vegetation seen.

Supplemental Table 3: Overview of clinical vignette patient's subsequent diagnostic workup
