# Supplementary figures and images for: Multimodality Imaging in the Diagnosis of Prosthetic Valve Endocarditis: A Brief Review
Source: Front Cardiovasc Med. 2021 Dec 20;8:750573. doi: 10.3389/fcvm.2021.750573 (PMC8720921; doi:10.3389/fcvm.2021.750573)

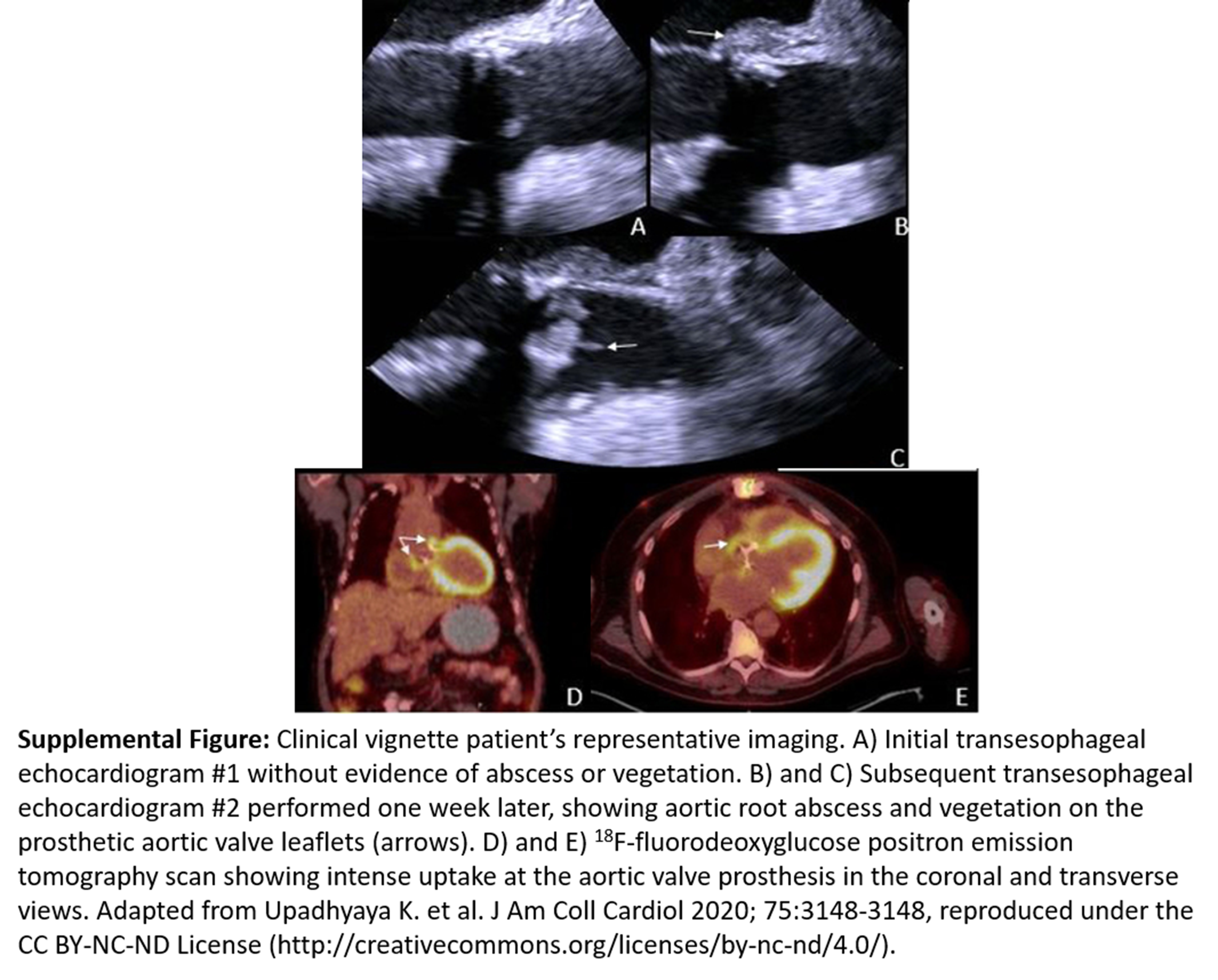

Supplement: Supplementary file 4 [file Image_1.tif]
